# Clinical Selection of Prenatal Diagnostic Techniques Following Positive Noninvasive Prenatal Screening Results in Southwest China

**DOI:** 10.3389/fgene.2021.811414

**Published:** 2022-01-28

**Authors:** Xiaosha Jing, Hongqian Liu, Qian Zhu, Sha Liu, Jianlong Liu, Ting Bai, Cechuan Deng, Tianyu Xia, Yunyun Liu, Jing Cheng, Xiang Wei, Lingling Xing, Yuan Luo, Quanfang Zhou, Lin Chen, Lingping Li, Jiamin Wang

**Affiliations:** ^1^ Department of Obstetrics and Gynaecology, West China Second University Hospital, Sichuan University, Chengdu, China; ^2^ Key Laboratory of Birth Defects and Related Diseases of Women and Children, Ministry of Education, Sichuan University, Chengdu, China

**Keywords:** aneuploidy, noninvasive prenatal screening, sex chromosome mosaicism, other chromosomal abnormalities, positive NIPS results, prenatal diagnostic techniques, sex chromosome aneuploidy

## Abstract

**Background:** This study aims to evaluate prenatal diagnosis methods following positive noninvasive prenatal screening (NIPS) results.

**Methods:** According to the positive noninvasive prenatal screening results, 926 pregnant women were divided into three groups: main target disease group (high risk for trisomy 21, trisomy 18, or trisomy 13), sex chromosome aneuploidy (SCA) group, and other chromosomal abnormalities group [abnormal Z-scores for chromosomes other than trisomy (T)21/T18/T13 or SCAs]. The verification methods and results were then retrospectively analysed.

**Results:** In the main target disease group, the positive rate of chromosomal abnormalities confirmed by quantitative fluorescence polymerase chain reaction (QF-PCR) was 75.18% (212/282), which was not significantly different from that by karyotyping (79.36%, 173/218) and copy number variation (CNV) detection methods (71.43%, 65/91). The positive rate of additional findings confirmed by karyotyping and copy number variation detection methods in main target disease group was 0.46% (1/218) and 8.79% (8/91), respectively. The positive rate of chromosomal abnormalities confirmed by karyotyping and CNV detection methods were 27.11% (45/166) and 38.46% (95/247) in the SCA group and 4.17% (1/24) and 20% (36/180) in the other chromosomal abnormalities group, respectively. Fetal sex chromosome mosaicism was detected in 16.13% (20/124) of the confirmed SCA cases. There were no significant differences in the detection rates of chromosomal microarray analysis (CMA) and CNV sequencing (CNVseq) among the three groups (*p* > 0.05).

**Conclusion:** QF-PCR can quickly and accurately identify aneuploidies following NIPS-positive results for common aneuploidy, and in combination with karyotyping and CNV detection techniques can provide more comprehensive results. With the NIPS-positive results for SCA or other abnormalities, CMA and CNVseq may have the same effect on increasing the detection rate. The addition of fluorescence *in situ* hybridization assay may help to identify true fetal mosaicism.

## Introduction

Noninvasive prenatal screening (NIPS) based on cell-free DNA in maternal circulation is used primarily to screen for trisomies 21, 18, and13, that has been shown to outperform conventional screening methods (Norton et al., 2015). In addition, NIPS may reveal other chromosomal abnormalities, such as sex chromosome aneuploidy (SCA) and other autosomal aberrations, by low-coverage whole-genome sequencing of maternal and placental fragments of DNA (Nicolaides et al., 2013; Lefkowitz et al., 2016; van der Meij et al., 2019). Not all positive results detected by NIPS represent fetal aberrations because of certain biological factors, such as confined placental mosaicism (Grati, 2014; Grati, 2016), ‘vanishing’ twins (Grömminger et al., 2014), and maternal genomic contribution (Ji et al., 2018; Zhang et al., 2020). Hence, further prenatal diagnostic testing is required to check NIPS-positive results.

Several frequently used prenatal diagnostic technologies vary in the testing principle, turnaround time, detection range, and cost. Cytogenetic karyotype testing enables the visual detection of chromosomal abnormalities such as full chromosome aneuploidies, polyploidy, mosaicism, and structural abnormalities of more than 5–10 million base pairs (Mb), including balanced and unbalanced rearrangements. Both quantitative fluorescence polymerase chain reaction (QF-PCR) and fluorescence *in situ* hybridization (FISH) provide a rapid prenatal diagnosis of chromosome aneuploidy, and FISH is a valuable complementary test for detecting mosaicism (Hultén et al., 2003; Su et al., 2015). Chromosomal microarray analysis (CMA) and copy number variation sequencing (CNVseq) assays, as the two major CNV detection techniques, offer a high-resolution approach to diagnose microdeletions or microduplications (Xu et al., 2014; Wang et al., 2020). CMA can be divided into two types: array-based comparative genomic hybridization (aCGH) and single nucleotide polymorphism (SNP) array.

During recent years, NIPS testing has considerably increased. While most studies have focused on the detection range and accuracy of NIPS(Nicolaides et al., 2013; Lefkowitz et al., 2016; Deng et al., 2019; van der Meij et al., 2019), only a few have investigated prenatal diagnostic techniques following positive NIPS results. In this study, we retrospectively analysed the prenatal diagnostic methods following different types of NIPS-positive results. This study, to the best of our knowledge, the first of its kind in southwest China, aims to provide more evidence for the clinical selection of prenatal diagnosis in patients with different types of NIPS-positive results.

## Methods

### Participants

This retrospective analysis involved 926 pregnant women with positive NIPS results who underwent amniocentesis between May 2015 and June 2019 at West China Second University Hospital of Sichuan University, Chengdu, Sichuan Province, China.

The NIPS-positive results were classified into three categories: 1) “main target disease” if NIPS was positive for trisomy (T)21, T18, or T13; 2) “SCA” when NIPS was positive for SCA; and 3) “other chromosomal abnormalities” when NIPS presented abnormal Z-scores for chromosomes other than T21/T18/T13 or SCAs. All participants received professional pre-test counselling before NIPS testing and amniocentesis. They all voluntarily chose to expand NIPS to obtain maximum fetal genomic information; and then selected one or more diagnostic methods for the ultimate report from karyotyping, CMA, and CNVseq, after knowing the scope of application, target diseases, limitations of these prenatal diagnostic techniques, and decided whether rapid testing (QF-PCR/FISH) would be performed. Statistical analyses of the diagnostic methods and results were conducted. Neonatal conditions of those diagnosed as negative by amniocentesis were followed up for growth and development via phone call.

The study was approved by the Institutional Ethics Committee of Sichuan University and conducted in accordance with the Declaration of Helsinki. All participants signed a written informed consent form before testing.

### Noninvasive Prenatal Screening

Maternal peripheral blood (10 ml) was collected in a cell-free BCT tube (Streck, Omaha, NE, United States). The procedural details of the experimental operation and analysis methods have been published previously (Liu et al., 2021). The test results for all 24 chromosomes were presented using Z-scores (normal range, −3 < Z < 3).

### Invasive Prenatal Diagnosis

Following written informed consent and performing a preoperative examination, 25–30 ml of amniotic fluid was collected by abdominal amniocentesis using ultrasonography guidance. The amniotic fluid was tested following strict instructions regarding the operation conditions and procedures for the corresponding diagnostic tests.

### Chromosome Karyotyping

Upon standard metaphase conversion of cultured fetal cells, amniotic fluid cells were cultured using BIOAMFTM-3 medium (Biological Industries, Kibbutz Beit-Haemek, Israel) and AMINOPAN medium (PAN-biotech GmbH, Aidenbach, Germany). Each cultured cell was inoculated in parallel with the two bottles. More than 20 metaphase cells from each specimen were analysed by g-banding at a resolution of above 400 bands on average. Karyotypes were recorded as text written in the International System for Human Cytogenetic Nomenclature (ISCN) 2020.

### QF-PCR

DNA was extracted from 5 ml of uncultured amniotic fluid samples using the QIAamp DNA Mini kit (QIAGEN, Dusseldorf, Germany). The concentration was adjusted to 30 ng/μL. PCR amplification was performed using a reaction kit (Guangzhou Darui Biotechnology Co., Ltd., Guangzhou, China). The PCR product and GeneScan™600 LIZ Size standard v2.0 was denatured, and electrophoretic analysis was performed using a POP4 gel (ABI) on the ABI 3500Dx Genetic Analyser (Applied Biosystems). There were four short tandem repeat (STR) markers for the X and Y chromosomes (AMXY, DXS981, DXS6809, DXS22), three for chromosome 21 (D21S1435, D21S11, D21S1411), four for chromosome 18 (D18S1002, D18S391, D18S535, D18S386) and four for chromosome 13 (D13S628, D13S742, D13S634, D13S305). Data collection and analysis were conducted using Gene Mapper ID Software V3.2 (Applied Biosystems, Inc., CA, United States).

### FISH

The uncultured amniotic fluid was tested, following strict instructions of the operation conditions and procedure, using the AneuVysion Multicolor DNA Probe kit (Abbott Molecular, Inc., Des Plaines, United States), according to the manufacturer’s instructions. Two sets of CEP 18/X/Y and LSI 13/21 probes were used, which are located on the centromere of chromosome 18/X/Y, 13q14, and 21q22.13-q22.2. After hybridising the fluorescent-labelled DNA probe with the sample DNA, the fluorescence microscope OLYMPUS BX51 was used to assess chromosome aneuploidy.

### CMA

The American Cytoscan®Affymetrix 750 K chip kit and reagent kit bundle (Beijing Guoku Biological Technology Co., Ltd., Beijing, China), and the Clontech Titanium DNA Amplification kit (Clontech, United States) were used for DNA extraction, fragmentation, and hybridization. Hybridization products were then washed, stained on Fluidics Station 450Dx v.2, and subsequently scanned on Gene Chip Scanner 3000Dxv.2 with Auto Loader. The obtained data were loaded into Chas V3.0 software for data processing.

### CNVseq

DNA was extracted from amniotic fluid using a DNA extraction test kit (Hangzhou Berry Gene Diagnostic Technology Co., Ltd., Hangzhou, China). This was followed by DNA fragmentation, library construction using PCR-free technology, and index addition. The quality of the libraries was tested using the KAPA SYBERFAST qPCR kit (KAPA Biosystems, Wilmington, MA, United States) before and after pooling the libraries. The hybrid library was then subjected to 36 bp single-terminal sequencing with a sequencing depth of 0.1× using a Next Seq CN500 massively parallel sequencing kit (High-throughput sequencing kit) and the NextSeqCN500 high-throughput sequencing flow cell with four lanes (Illumina China, Shanghai, China). The sequences obtained were compared with those of the hg19 human genome.

### Classification Criteria for CNVs

The results were determined by referring to the hg19 version of the human genome and the most recent data available on the Database of Genomic Variants, DECIPHER, Online Mendelian Inheritance in Man, University of California Santa Cruz, PubMed, and other public databases. According to the American College of Medical Genetics (ACMG) sequence variant classification guidelines for copy number variants (Richards et al., 2015), the clinical significance of CNVs was divided into five classes: pathogenic, likely pathogenic, benign, likely benign, and variant of uncertain significance (VUS).

### Statistical Analysis

SPSS software v21 (IBM, Armonk, NY, United States) was used to analyse the data. Enumeration data are expressed as example and percentage (%). The chi-square (χ^2^) test was used to compare the difference in detection rates between the two methods. For a total number of <40 or a theoretical frequency <5, Fisher’s exact probability method was used. A *p* value of <0.05 was considered significant.

## Results

According to the NIPS results, patients were divided into three groups: main target disease [T21 (*n* = 234), T18 (*n* = 44), and T13 (*n* = 31)], fetal sex chromosome abnormalities (*n* = 413), and other chromosomal abnormalities (*n* = 204) groups. The average maternal age, average gestational age, and true positive rate of each group are summarized in Table 1. In the main target disease group, 240/309 (77.70%) were confirmed as chromosome abnormalities including 233/240 (97.08%) with common trisomy (two of which were combined with other abnormalities) and 8/240 (3.33%) with CNV. In the SCAs group, 140/413 (33.90%) were confirmed as chromosome abnormalities including 115/140 (82.14%) with sex chromosome aneuploidy (one of which was combined with autosome CNV), 26/140 (18.57%) with only CNV and one with structural abnormality. In the other chromosomal abnormalities group, 37/214 (17.29%) were confirmed as chromosome abnormalities including 2/37 (5.41%) with chromosome aneuploidy, 28/37 (75.68%) with CNV and 7/37 (18.92%) with absence of heterozygosity (AOH). The NIPS exception types, diagnostic methods, and results of amniocentesis are presented in Table 2.

**TABLE 1:** Summary statistics of 926 patients with positive NIPS results.

NIPS result	Patients (N)	Positive rate of aneuploidy (%)	Maternal age in years (mean, range)	Gestational age in weeks at NIPS (mean)
Main target diseases	309	77.67	31 (19–44)	17
Sex chromosome aneuploidy	413	33.90	29 (18–43)	18
Other chromosome abnormalities	204	18.14	29 (18–42)	18

**TABLE 2:** Comparison of positive rates by different detection methods for the cases with different types of NIPS-positive results.

NIPS result	Karyotype			CNV detection technologies						Karyotype vs. CNV detection technologies	CMA vs. CNVseq
				CNVseq			CMA				
	Aneuploidy	Structural abnormalities	True positives/test cases (%)	Aneuploidy	CNV	True positives/test cases (%)	Aneuploidy	CNV+AOH	True positives/test cases (%)		
Main target diseases	173	1 (0.46%)	173/218^a^ (79.36%)	50^b^ (65.79%)	8^b^ (10.53%)	57/76 (75%)	8 (53.33%)	None	8/15 (53.33%)	0.133	0.119
SCA	44 (26.51%)	1 (0.60%)	45/166 (27.11%)	14 (26.92%)	None	14/52 (26.92%)	57^c^ (29.23%)	27^c^ (13.85%)	81/195 (41.54%)	0.02	0.077
Other chromosome abnormalities	1 (4.17%)	None	1/24 (4.17%)	None	5 (14.29%)	5/35 (14.29%)	1 (0.69%)	30 (20.69%)	31/145 (21.38%)	0.086	0.481

a Two cases (cases 107 and 212) had test failure with karyotype analysis and were diagnosed as aneuploidy by QF-PCR.

b One case was diagnosed with trisomy 21 combined with CNV.

c Three cases were diagnosed with sex chromosome aneuploidy combined with autosome CNV.

SCA, sex chromosome aneuploidy; CNV, copy number variation; CNVseq, copy number variation sequencing; CMA, chromosomal microarray analysis.

### Prenatal Diagnosis of Cases With Positive NIPS Result for the Main Target Disease

A common trisomy was found in 233 (75.40%) cases (T21, 198; T18, 30; and T13, 5), including five cases of mosaic trisomy (three mosaic T21, one mosaic T18, and one mosaic T13) and five cases of robertsonian translocation [three 46,X?,rob(14;21)(q10;q10),+21, one 46,X?,rob(15;21)(q10;q10),+21, and one 46,X?,+21,rob(21;21)(q10;q10)]. Additional findings of nine cases are listed in Table 3. The levels of other exceptions detected ranged from 0.46%(1/218) to 8.79% (8/91) based on results of karyotyping and CNV detection technologies, respectively. Of these, 66.67% involved chromosomes with abnormal *Z*-values (*n* = 6) and 33.33% involved other autosomes (chromosomes 2 and 3). A total of 282 patients in the main target disease group underwent the QF-PCR test. Compared with the final reports, the QF-PCR assay correctly identified all 207 cases of target aneuploidies (178 T21, 25 T18, and 4 T13), including five mosaics, and helped confirm aneuploidies in two cases (cases 107 and 212) with culture failure. The QF-PCR result of case 282 showed that both D13S628 and D13S634 markers illustrated triallelic patterns, while the two markers D13S742, and D13S305 exhibited clear heterozygous patterns within the normal range. The karyotype of the pregnant woman was normal, and the karyotype of the fetal father was 46,XY,t(12;13)(q24;q14). No significant difference was observed in the positive rate between QF-PCR and karyotyping (*p* = 0.381), QF-PCR and CNV detection techniques (*p* = 0.330), karyotyping and CNV detection techniques (*p* = 0.133), and CNVseq and CMA (*p* = 0.119) for the main target disease group.

**TABLE 3:** Additional findings by amniocentesis in 9 cases with positive NIPS for main target diseases.

Case	NIPS results	Z-scores	QF-PCR results	Final report		Pathogenicity classification
				Methods	Result	
258	T13	5.64	Abnormal signal at chromosome 13 site	Karyotype	46,XX,add(12)(q24)	
132	T13	5.6	(—)	CNVseq	Seq[hg19] 13q12.12q12.12 (23560000-24900000)x3 (1.34 Mb)	LB
137	T18	3.53	(—)	CNVseq	Seq[hg19] 2p25.3p25.3 (0-2380000)x1 (2.38 Mb)	P
190	T18	3.24	Abnormal signal at chromosome 18 site	CNVseq	Seq[hg19] 18p11.31p11.23 (3920000-8080000)x3 (4.16 Mb)	VUS
194	T21	3.02	(—)	CNVseq	Seq[hg19] 21q21.1 (9080000-19980000)x3 (0.90 Mb)	VUS
252	T18	3.65	(—)	CNVseq	Seq[hg19] 2q21.1q21.2 (132180000-133480000)x3 (1.30 Mb)	VUS
295	T21	3.54	(—)	CNVseq	Seq[hg19] 21q21.1q21.2 (23280000-25100000)x3 (1.82 Mb)	VUS
323^a^	T18	3.53	Abnormal signal at chromosome 18 site	CNVseq	1. Seq[hg19] 13q14.3q21.1 (54380000_55580000)x1 (1.20 Mb); 2. Seq[hg19] 18q22.1q22.1 (63880000_66580000) x3 (2.70 Mb)	1. VUS; 2. VUS
326	T21	8.14	T21	CNVseq	21T,dup3p14.3p14.2(57960000-59120000)x3 (1.16 Mb)	VUS

NIPS, non-invasive prenatal screening; QF-PCR, quantitative fluorescence polymerase chain reaction; T21, trisomy 21; T18, trisomy 18; T13, trisomy 13; P, pathogenic; LP, likely pathogenic; VUS, variants of uncertain significance; LB, likely benign.

a Case 323: Karyotype analysis results of amniotic fluid were negative.

### Prenatal Diagnosis of Cases With Positive NIPS Result for SCA

The positive karyotyping rate was lower than that of the CNV detection methods (*p* = 0.02). One hundred and fifteen (27.85%) cases were confirmed as sex chromosome aneuploidies by amniocentesis (two cases of monosomy X, 22 of XXX, 47 of XXY, 24 of XYY, one of XXYY, and 19 of sex chromosome mosaicism). The 19 cases with amniotic fluid findings involving sex chromosome mosaicisms are summarized in Table 4. There were eight other cases confirmed to involve sex chromosome abnormalities, including one sexual chromosome structural abnormality [45,X,del(X)(q24)] and seven sex chromosome CNVs [four were classified as pathogenic CNV (pCNV)]. Additionally, 20 fetuses were confirmed to have autosomal CNVs. Among the 27 CNVs found by CNV detection methods, eight were classified as pCNV or likely pCNV as shown in Table 5, including two CNVs with an abnormal size of >5 Mb and six with an abnormal size of <5 Mb. The other 19 CNVs with an abnormal size of <5 Mb were identified as two likely benign and 17 VUS.

**TABLE 4:** Distribution of sex chromosome mosaicism diagnosed prenatally.

Case	NIPS results	Final report		FISH results
		Methods	Results	
547	X0	Karyotype	45,X[19]/46,XX[11]	X[45]/XX[55]
548	X0	Karyotype	45,X[14]/46, XX[9]	X[29]/XX[71]
557	X0	Karyotype	45,X[6]/46,XY[27]	X[9]/XY[91]
588	X0	Karyotype	45,X [16]/46,XX [5]	X
680	X0	Karyotype	45,X[9]/46,XX[11]	X[32]/XX[68]
765	X0	Karyotype	46,X,del(X)(P21)[23]/45,X[6]/46,XX[5]	Declined
838	X0	Karyotype	45,X[15] /47,XXX[12]	Declined
984	X0	Karyotype	47,XXX[6]/46,XX[24]	Declined
561	X0	Karyotype	45,X	XXX[50]/X[48]/XX[2]
568	X0	Karyotype	45,X	X[30]/XYY[13]/XY[57]
648	XXY	CMA	arr[hg19] X*1.25, Y*1	XXY[47]/XY[53]
695	X0	CMA	arr[hg19] X*1.77, Y*0	X[12]/XX[88]
767	X0	CMA	arr[hg19] X*1.33, Y*0	X[57]/XXX[39]/XX[4]
787	X0	CMA	arr[hg19] X*1.20, Y*0	XXX[91]/X[9]
814	X0	CMA	arr[hg19] X*1.63, Y*0	X[45]/XXX[55]
826	XXY	CMA	arr[hg19] X*1.33, Y*1	XXY[42]/XY[58]
923	X0	CMA	arr[hg19] X*1.80, Y*0	XXX[30]/X[8]/XX[62]
925	X0	CMA	arr[hg19] X*1.32, Y*0	X[28]/XX[72]
983	X0	CNVseq	seq[hg19] X*1.20, Y*0	X[52]/XX[48]

NIPS, non-invasive prenatal screening; CMA, chromosomal microarray analysis; CNVseq, copy number variation sequencing.

**TABLE 5:** Likely pathogenic and pathogenic CNVs by CNV detection techniques in 8 cases with positive NIPS for SCA.

Case	NIPS result	prenatal diagnostic techniques	Result	Pathogenicity classification
738	X0	CMA	arr[hg19] 7q36.1q36.3 (152364657-159119707)x1 (6755 kb)	P
750	X0	CMA	1. arr[hg19] 19q13.42q13.43 (54718954-58956816)x3 (4238 kb)	1.LP; 2.P
			2. arr[hg19] Xq21.1q28 (83949636-155233098)x1 (71283 kb)	
790	X0	CMA	arr[hg19] Xp22.31 (6455151-8141076)x1 (1686 kb)	P
809	X0	CMA	1. arr[hg19] Xp22.33p11.21 (168551-57033787)x1 (56865 kb)	1.P; 2.P
			2. arr[hg19] Xp11.21q28 (57037147-155233098)x3 (98196 kb)	
823	X0	CMA	arr[hg19] 3q29 (195902886-197386180)x3 (1483 kb)	LP
825	X0	CMA	arr[hg19] 21q22.3 (47692007-48093361)x1 (401 kb)	LP
848	XXX	CMA	1. arr[hg19] Xp22.33q28 (168551_151841206)x3 (151673 kb)	1.P; 2.P
			2. arr[hg19] Xq28 (151859630_155233098)x1 (3373 kb)	
966	XXX	CMA	1. arr[hg19] 5q23.1 (115241753-115737646)x3 (496 kb); 2. arr[hg19] 16p11.2 (29581101-30190029)x3 (609 kb)	1.VUS; 2.P

Moreover, a sex chromosome mosaicism (case 840, Table 7) confirmed by peripheral karyotyping of the newborn was revealed upon follow-up investigation. The positive prediction value (PPV) of NIPS was 28.09% (116/413) for detecting fetal SCAs.

### Prenatal Diagnosis of Cases With Positive NIPS Result for Other Chromosomal Abnormalities

Of 204 cases with positive NIPS result for other autosomal abnormalities, amniocentesis confirmed 37 cases with chromosomal abnormalities and only 23 with results corresponding to that of NIPS. The remaining 14 cases were confirmed with additional discoveries <5 Mb, including three pCNVs. The overall PPV was 11.27% (23/204); however, this percentage differed per chromosome. CNV detection techniques identified 28 cases of CNV, seven cases of AOH, and one T16 mosaicism. Of the total observed CNVs, 57.14% (16/28) involved CNVs with an abnormal size of <5 Mb, including three classified as pCNV, 12 as VUS, and 1 as likely benign. Furthermore, 42.86% (12/28) involved CNVs with an abnormal size of >5 Mb, which were classified as follows: six of pCNV, two of likely pathogenic (LP), and four of VUS. Likely pathogenic and pathogenic CNVs in 11 cases were shown in Table 6. One of seven AOHs was diagnosed as maternal uniparental disomy of chromosome 6 [upd (6)mat], following further testing of the parental samples. One case of mosaicism (47,XX,+9 [4]/46,XX [16]) was detected in 24 patients who underwent karyotyping.

**TABLE 6:** Likely pathogenic and pathogenic CNVs by CNV detection techniques in 11 cases with positive NIPS for other chromosomal abnormalities.

Case	NIPS results		Prenatal diagnosis		
	Chromosome	Z-scores	Methods	Result	Pathogenicity classification
335	Chr21	−35.77	CMA	arr[hg19]21q11.2q21.3(15016486-29188153)x1(14172 kb)	LP
365	Chr7	12.83	CMA	arr[hg19]Xp22.31(6449836-8143509)x1(1694 kb)	P
394	Chr16	−3.29	CMA	arr[hg19]16p13.3p12.3(12548051-18242713)x1(5695 kb)	P
416	Chr9	12.63	CMA	1.arr[hg19]5p15.33(113578-1641941)x1 (1528kb); 2. arr[hg19]9p24.3p22.3(208454-15698373)x3 (15400 kb)	1.VUS; 2.P
429	Chr3	5.98	CMA	arr[hg19] 3q27.2q29(185409996_197851444)x3 (12441 kb)	P
442	Chr20	3.81	CMA	1. arr[hg19]11q24.2q25(126392021_134937416)x1 (8545kb); 2. arr[hg19] 20p13p11.21(61661_24487341)x3 (24426 kb)	1.VUS; 2.P
482	Chr14	−4.26	CMA	arr[hg19] Yq11.21q11.221 (14460771_15220682)x0 (760 kb)	LP
496	Chr5	3.01	CMA	arr[hg19] 2q37.3 (239034152_242782258)x1 (3748 kb)	P
507	Chr5	−3.53	CMA	arr[hg19] 5p15.33p14.1 (113576_26243789)x1 (26130 kb)	P
522	Chr3	3.2	CMA	1.arr[hg19] 3p26.3p22.2 (285856_37597219)x3 (37311 kb)	1.P; 2.P
				2.arr[hg19] 5p15.33p15.31 (113576_8750244)x1 (8637 kb)	
383	Chr3	−4.21	CNVSeq	seq[hg19] 3q13.32q21.2 (117980000-124720000)x1 (6740 kb)	LP

### Follow-up Investigation of 508 Cases With Initial Positive NIPS Results and Subsequent Negative Results in Amniocentesis

A total of 125 cases were lost to follow-up, and eight cases terminated the pregnancy due to complications during pregnancy, such as hypothyroidism and gestational hypertension. Among the 383 cases that were followed up, seven abnormal newborns were observed (Table 7). One abnormal newborn with perineal urethral stenosis underwent peripheral karyotyping and was diagnosed with a sex chromosome mosaicism (45,X [24]/46,XY [36]). Through the appraisal of growth and development by senior pediatricians for the rest neonates, there were no T21, T18, or T13; and no SCA or related abnormalities were reported during neonatal examination and follow-up.

**TABLE 7:** Details of abnormal newborns with negative prenatal diagnoses.

Case no.	NIPS results	Diagnostic methods	Neonatal abnormalities	Peripheral karyotype of newborn
336	Trisomy 7	Karyotype	Syndactyly	Normal
373	Trisomy 9	CNVseq	Congenital heart disease	Refused
391	Trisomy 2	CMA	Ventricle septal defect	Refused
642	X0	Karyotype	Hearing impairment	Refused
666	XXY	CNVseq	External ear malformation	Refused
782	ChrX-(Y)	Karyotype	All internal organs reverse	Refused
840	X0	CMA	Perineal urethral stenosis	45,X[24]/46,XY[36]

CMA, chromosomal microarray analysis; CNVseq, copy number variation sequencing.

## Discussion

With the popularisation of medical knowledge, prenatal testing has been gaining increasing attention. The ACMG emphasises that detailed pre-test counselling and obtaining informed consent of pregnant women are the basis for expanding the scope of NIPS(Gregg et al., 2016). For an increasing number of pregnant women, NIPS is becoming an option to achieve high-resolution, sensitive, and specific detection of common trisomy and additional findings (van Schendel et al., 2015; van der Meij et al., 2019), as confirmed in this study. The PPV of NIPS was 75.40% for the main target disease, 28.09% for SCA, and 11.27% for other abnormalities, which was slightly lower than that predicted by previous studies (Norton et al., 2015; Deng et al., 2019; van der Meij et al., 2019). This might be relate to the fact that only pregnant women undergoing amniocentesis were included in this study.

Karyotyping is the most common validation method for positive NIPS results in many laboratories, followed by CMA, according to previous reports (Dobson et al., 2016; Cherry et al., 2017; Xu et al., 2020). Several problems have been identified in the actual application process. Culturing amniotic fluid cells and G-banding analysis make karyotyping time-consuming and requiring technical expertise; moreover, the long reporting period leads to anxiety in patients. In addition, karyotyping cannot reliably detect genomic segment rearrangements of <5–10 Mb. CMA can detect changes as small as 5–10 kb in size; however, its low throughput and high cost are obvious drawbacks.

Owing to the high PPV of NIPS for the main target disease, a rapid validation result is significant for pregnant women, and it could reduce parental anxiety and improve pregnancy management (Hultén et al., 2003; Ju et al., 2021). QF-PCR can be used to detect common aneuploidies within 24–48 h. When used alone, it helps to reduce parental anxiety and healthcare cost (Hills et al., 2010; de la Paz-Gallardo et al., 2015). Consistent with other clinical research reports (Voglino et al., 2002; Nicolini et al., 2004), the coincidence rate of QF-PCR and karyotyping in the diagnosis of common trisomies in this study was 100%. The results of QF-PCR did help these pregnant women to plan the next steps of pregnancy management more quickly. There has been controversy over whether QF-PCR can replace karyotyping as a stand-alone method in the general pregnant population, because a residual risk exists for clinically significant chromosomal abnormalities associated with normal QF-PCR results. QF-PCR is used in prenatal diagnostics exclusively for the analysis of numerical changes. Herein, the miss rate of QF-PCR tests at the karyotype detection level was 0.32% (1/309)—slightly higher than 0.069–0.12% reported in several studies with a large number of patients (Comas et al., 2010; Hills et al., 2010; Papoulidis et al., 2012); one of the primary causes for this result was the relatively small number of cases in our study. Theoretically, NIPS has a wider screening range and lower omission rate than traditional screening, which should translate to a lower residual risk of clinically significant chromosomal abnormalities associated with normal QF-PCR results in the main target disease group; however, further research is required to confirm this.

In some cases in the main target disease group, additional findings by karyotyping and CNV detection methods provided important information for effective genetic counseling. Robertsonian translocation accounts for approximately 2.31% (5/216) of all Down syndrome cases diagnosed by karyotyping in our study. QF-PCR is unable to distinguish the complete trisomy 21 from the trisomy as a consequence of translocation of chromosome 21, that is unlikely to affect present pregnancy management; however, it may affect the assessment of risk of recurrence. In case 258, considering the QF-PCR result (D13S628 and D13S634 markers both indicate the triallelic patterns) and karyotyping result (46,XX,add (12)(q24)), it suggested that there may be a balanced translocation carrier in fetal parents. Fetal father was identified as a carrier by further karyotyping, which provided an important basis for assessing the risk of offspring with an unbalanced structural abnormality. In a situation like this, karyotyping was a basic technique which cannot be replaced by other prenatal diagnostic techniques. Using CNV detection technology, we found that 6.59% (5/91) had corresponding chromosome microduplication rather than full trisomy, including case 190 and case 323 with ambiguous QF-PCR results. These findings demonstrated that submicroscopic duplications may result in NIPS showing a high-risk of trisomy, and CNV detection methods can aid in the clarification of abnormalities detected by NIPS. Furthermore, CNV detection technology has a 1.10% (1/91) higher yield of pathogenic CNVs than traditional karyotype analysis in women with positive NIPS resut for T21/T18/T13; this is in line with the findings in the general population of pregnant women (Wang et al., 2018). CNV detection techniques increased additional positive results for fetuses with trisomy 21/18/13 suspected by NIPS; however, the majority of additional findings (75%, 6/8) were classified as VUS, which increased the difficulty in genetic counselling and caused anxiety in pregnant women.

Here, the positive rate of the chromosomal aberration in the SCA group detected by karyotyping was lower than that by the CNV detection methods (*p* = 0.02), thus confirming that the performance of karyotyping is lower than that of CNV detection methods in confirming the SCAs predicted by NIPS. Sund et al. reported additional risks for structural abnormalities of sex chromosomes in fetuses with a noninvasive prenatal screen positive for SCA(Sund et al., 2020). A recent study has shown that the CNV detection method may provide additional findings for women with positive NIPS results for fetal SCA(Xu et al., 2020). In this study, 2.43% (6/247) fetuses in the SCA group were diagnosed with pCNV or likely pCNV (<5 Mb) by CNV detection methods (Table 5). This further highlights that the CNV detection technique can increase the diagnostic yield for women with positive NIPS result for SCA and should be recommended. In the other chromosomal abnormalities group, the detection rates of CNV detection methods and karyotyping were not significantly different. Nevertheless, caution should be exerted when interpreting the lack of statistical significance, as a relatively small number of people chose karyotyping in our study. Based on the assumption that the resolution of karyotyping is on the order of 5–10 Mb, 8.89% (16/180) anomalies diagnosed as <5 Mb by the CNV detection method would be missed by karyotyping in the other chromosomal abnormalities group, including 1.67% (3/180) classified as pathogenic CNV. Furthermore, there were seven (4.83%) cases of AOH identified by CMA, including one (0.69%) that was ultimately diagnosed with uniparental disomy (UPD). AOH provides clues to explore recessive diseases, and UPD for a clinically relevant chromosome (chromosomes 6, 7, 11, 14, 15, or 20) can result in abnormal phenotypes (Shaffer et al., 2001). The corresponding trisomy associated with UPD, which usually presents only in the placenta, can result in an abnormal NIPS Z-scores (Van Opstal et al., 2018). The ACMG statement indicated that any imprinted regions or chromosomes reportedly involved in positive NIPS cases with discordant results should consider undergoing specific UPD analyses (Cherry et al., 2017).

In the past few years, CMA has been widely recommended as the first-tier test for high-risk pregnancies to identify microscopic/submicroscopic CNVs(Chung et al., 2020; Wang et al., 2020). However, its low throughput, high cost, and complicated operation hinder widespread use. CNVseq based on massively parallel sequencing has several beneficial features, such as low cost, high throughput, low demand of DNA (10 ng), and high resolution (0.1 Mb). CNVseq has been demonstrated to be a suitable first-tier diagnostic test for the identification of clinically significant fetal chromosome anomalies, and it has shown similar effectiveness and advantages in the clinical diagnosis of aneuploidy and pCNVs(Ma et al., 2021). CNVseq could even detect some CNVs that were omitted by CMA because of insufficient probes within the corresponding regions and showed a higher sensitivity in detecting low-level mosaicism than CMA (Walker et al., 2019; Wang et al., 2020). Herein, CMA and CNVseq showed no significant differences in the positive rate in the three types of abnormalities indicated by NIPS, which further indicate that CNVseq represents an economic alternative to increase the abnormality detection rate in pregnant women with positive NIPS results. However, CNVseq cannot fully replace CMA. Except for aneuploidy, chromosomal CNV, and mosaicism, CMA based on the SNP platform can identify AOH and UPD; hence, cases with imprinted chromosome abnormalities predicted by NIPS may benefit more from CMA. However, neither CMA nor CNVseq can identify balanced translocations, and neither aCGH array nor CNVseq can identify triploidy. Neither of these chromosomal abnormalities can be detected by NIPS. Thus, karyotyping remains a valuable method for prenatal diagnosis and may provide unexpected yields for NIPS-positive pregnant women.

Additionally, the sex chromosome mosaicism was relatively frequently diagnosed in this study, accounting for 16.13% (20/124) of cases in the group with confirmed sex chromosome abnormalities. The frequency was similar to the rate of 13.74% (18/131) in a review of large case series of NIPS(Xie et al., 2020). Accurate identification of fetal mosaicism has clinical importance in guiding genetic counselling. FISH detection, using targeted locus-specific probes to observe the chromosomes of each cell without cell culture, demonstrates unique advantages in identifying chromosomal mosaicism. FISH can identify true fetal mosaicism by detecting uncultured amniocytes. In addition, FISH can be used to detect placental mosaicism. Confined placental mosaicism is one of the main reasons for the inconsistency between NIPS results and prenatal diagnosis results (Grati, 2016). A reflex FISH test is recommended to confirm chromosome mosaicism when a mosaic pattern is highly suspected (Hultén et al., 2003; Cherry et al., 2017; Wang et al., 2018; Chai et al., 2019). Compared with the FISH results, we found a higher proportion of the 45,X counted by karyotyping. Besides the different number of cells analysed, better growth of the abnormal cell type is likely to be the major contributor. Culture and harvesting of amniotic fluid cells may lead to changes in the mosaic rate of different cell lines and even cause pseudo-mosaicism (Grati, 2014; Chai et al., 2019). Moreover, overgrowth by the X0 cell line might explain why the FISH results for cases 561 and 568 were inconsistent with those of karyotyping. Contrary to the previous two cases, the karyotyping of case 588 revealed mosaicism, whereas all 100 cells analysed by FISH were X0. Maternal cell contamination is recognized as the most likely interpretation. However, the chance of true fetal mosaicism cannot be completely ruled out. Compared with karyotyping, CNV detection techniques can test uncultured amniotic fluid cells, thus reducing the influence of cell culture on the mosaic ratio and restrictions of the cell cycle on prenatal diagnosis. However, owing to fluctuations in the fluorescence signal intensity and background noise, CMA has a low sensitivity to a lower proportion of mosaic patterns than karyotype analysis and CNVseq (Chai et al., 2019; Hao et al., 2020). The CMA result of amniotic fluid cell testing for case 840 was inconsistent with that of karyotyping of neonatal peripheral blood; the false negative result of CMA may be caused by the above reasons or tissue-specific chimerism. Previous studies have confirmed that CNVseq with increased sensitivity for mosaicism can accurately and reproducibly quantitate mosaicism at levels of 20% (Ruttanajit et al., 2016; Wang et al., 2020). However, neither CMA nor CNVseq can recognize fetuses with balanced mosaics made up of different cell lines (such as 47,XXX/45,X) (Markus-Bustani et al., 2012). Therefore, the FISH assay should additionally be performed to derive a more accurate karyotype and more accurately define the levels of sex chromosome mosaicism.

## Conclusion

For the majority of pregnant woman with a high-risk of common trisomy predicted by NIPS, QF-PCR stand-alone testing represents an economical and rapid method for prenatal diagnosis. Combined with karyotyping and CNV detection methods, some additional findings can be obtained, which can provide more comprehensive information for genetic counseling. When NIPS shows a positive result for SCA or other abnormalities, CNVseq can be employed as a valid and cost-effective alternative for CMA, which can improve the detection rate of pathogenic or potentially pathogenic chromosomal abnormalities. Considering the peculiarities of sex chromosomal mosaicism, the addition of the FISH assay can help identify true fetal mosaicism.

## Data Availability

The data analyzed in this study is subject to the following licenses/restrictions: The data for this article are not publicly available because of privacy concerns. Requests to access these datasets should be directed to the corresponding authors.
